# Color edges extraction using statistical features and automatic threshold technique: application to the breast cancer cells

**DOI:** 10.1186/1475-925X-13-4

**Published:** 2014-01-23

**Authors:** Salim Ben Chaabane, Farhat Fnaiech

**Affiliations:** 1 SIME Research Laboratory, ENSIT University of Tunis, 5 Av. Taha Hussein, 1008 Tunis, Tunisia

**Keywords:** Threshold, Statistical features, First order statistics, Second order statistics, Segmentation, Color edge detection

## Abstract

**Background:**

Color image segmentation has been so far applied in many areas; hence, recently many different techniques have been developed and proposed. In the medical imaging area, the image segmentation may be helpful to provide assistance to doctor in order to follow-up the disease of a certain patient from the breast cancer processed images. The main objective of this work is to rebuild and also to enhance each cell from the three component images provided by an input image. Indeed, from an initial segmentation obtained using the statistical features and histogram threshold techniques, the resulting segmentation may represent accurately the non complete and pasted cells and enhance them. This allows real help to doctors, and consequently, these cells become clear and easy to be counted.

**Methods:**

A novel method for color edges extraction based on statistical features and automatic threshold is presented. The traditional edge detector, based on the first and the second order neighborhood, describing the relationship between the current pixel and its neighbors, is extended to the statistical domain. Hence, color edges in an image are obtained by combining the statistical features and the automatic threshold techniques. Finally, on the obtained color edges with specific primitive color, a combination rule is used to integrate the edge results over the three color components.

**Results:**

Breast cancer cell images were used to evaluate the performance of the proposed method both quantitatively and qualitatively. Hence, a visual and a numerical assessment based on the probability of correct classification (P_*C*_), the false classification (P_*f*_), and the classification accuracy (*Sens(%)*) are presented and compared with existing techniques. The proposed method shows its superiority in the detection of points which really belong to the cells, and also the facility of counting the number of the processed cells.

**Conclusions:**

Computer simulations highlight that the proposed method substantially enhances the segmented image with smaller error rates better than other existing algorithms under the same settings (patterns and parameters). Moreover, it provides high classification accuracy, reaching the rate of 97.94%. Additionally, the segmentation method may be extended to other medical imaging types having similar properties.

## Introduction

Image segmentation is considered as a critical and an important basic operation for meaningful analysis and interpretation of image acquired [1]. It is one of the most difficult tasks in image processing, which determines the quality of the final results of analysis [2]. The goal of image segmentation is the decomposition of an image into meaningful or spatially coherent regions sharing similar features [3].

Color image segmentation attracts more and more attention in many pattern recognition and computer vision applications [4]. The additional information provided by color may be of great help in the image analysis process and yield better results than other approaches using only gray scale information. This situation may occur when the information cannot be extracted using only gray scale, then it becomes mandatory to use color information [3,4].

For instance, typical color image segmentation methods are mainly extended from monochrome segmentation approaches in different color spaces. In other words, gray level segmentation methods are directly applied to each component of a color space, and then the results are combined together to obtain the final segmentation results.

Different color image segmentation techniques have been, so far developed and detailed in the literature [3,4]. Generally, color image segmentation approaches are based on either discontinuity and/or homogeneity of information values in a region. The approach based on discontinuity tends to segment an image by detecting isolated points, lines and edges based on abrupt changes in gray levels. Typically, the approaches are usually based on homogeneity including threshold, clustering, region growing, region splitting and merging, etc. Consequently, the general color segmentation problem consists in choosing an adopted color model and the segmentation technique for a specific problem [4,5].

In the last decades; panoply of techniques have been proposed and studied solving the color edge detection problem [5-7]. Basically, these techniques detect the fast change between two regions [8]. Edge detectors used in these techniques turn out simple canny operator [8,9]. The output of most existing edge detectors can only provide candidates for the regions boundaries, because of the obtained color edges normally are discontinuous or over-detected. Whereas, in principle; the actual region boundaries should be closed curves. Consequently, some post-procedures, such as edge tracking, gap filling, smoothing, and thinning, should be performed in order to obtain the closed region boundaries. All these post-procedures are time-consuming; and converting the edge candidates to the region boundaries is thus not an easy task.

For instance, these time-consuming post-procedures may be avoided by integrating the results of the boundary-based approach over those + of the region based approach.

In a digital color image, a typical edge characteristic is defined when two brightness neighbouring pixels values change suddenly. Flowingly, in the literature many robust and complex color-edge detection techniques have been proposed [8,10,11]. These sophisticated techniques may provide highly accurate results.

These techniques are divided into two categories of edge detectors: 1) gradient operators and 2) second derivative operators.

Gradient operators, such as the Roberts, Prewitt, and Sobel operators [8], detect the edges by looking for the maximum and minimum in the first derivative of the luminance of an image.

The second derivative operators, such as the Marr-Hildreth and Marr-Poggio operators [11], search for zero-crossings in the second derivative of the luminance of an image to find the edges. However, most existing edge detectors consider only the horizontal and vertical edge patterns. In some applications of these methods, they suffer of drawbacks such time consuming, the obtained edges normally are discontinuous or over detected, and used only for gray level images.

The proposed color edge detection approach, in this paper, is conceptually different and explores a new strategy. In fact, instead of considering an elaborating a better designed color edge extraction model, the proposed technique rather explores the possible alternative of combining statistical features and the automatic threshold technique. After the determination of the features of each component image, the threshold technique is then used to extract the edge of each image to be combined. The feature images are computed on overlapping small windows centered on the pixel to be classified. Then, edge results for the three color components are integrated through a fusion rule in order to get a final reliable and accurate segmentation result. Hence, this work may be seen as a straightforward additional improvement of the issues proposed by Fan et al. [5].

For instance, this method is applied to color medical image segmentation, where our main purpose is to provide some additional help to the doctor to follow-up the state of patient disease breast cancer by enhancing and rebuilding some distorted and pasted cells to be easily counted. The objective is to rebuild each cell from a series of three component images (R, G and B). From an initial segmentation obtained by using statistical features and automatic threshold technique, one seeks a resulting segmentation which may represents as better as possible the cells, in order to show to the doctors an appropriate schema of the set of points really forming part of the cells, as well as a suitable representation of the cells to be easily counted and checked. Section 2 introduces the proposed method for color edge extraction. The experimental results are discussed in Section 3, and the conclusion is given in Section 4.

## Materials and methods

### Materials

In the medical domain, the doctors follow the diseases of the breast cancer taking into account the number of tumor cells expressing hormone receptor (HR). The assessment of labelled cells expressing the HR can be done on cells in suspension or histological over-part. This latter method is used in the pathology laboratory of cancer center Salah Azaiez in Tunis, Tunisia. The sample preparation is intended to highlight the different cells to be visible.

The cellular structure supports a procedure that requires several steps:

The fixing is used for the structures conservation and the parts hardening. It must be done immediately after sampling; by immersing the material in an adequate volume of fixative (the fixer used in Salah Institute Azaiez is the formalin at (10%)).

The inclusion allows realizing the fine and regular cuts. The most commonly used inclusion medium is the paraffin.

The cuts of the paraffin block are made with a microtome. They allow producing the section slices (thin slices) of 2 μm to 5 μm thick.

The coloring performed on slides; accentuates the contrasts for the better recognition of the different structure elements. Other colors are intended to highlight specific molecules such as the immunohistochemistry. In our study, the immunohistochemistry coloring is applied to highlighting the hormone receptors.

The installation: the colored cuts are placed between the blade and the cover slip with a synthetic refractive resin index close to that of glass. Then, a microscopic preparation is obtained ready to be observed with a microscope.

The acquisition is made by a digital camera NIKON Coolpix 995 mounted on a Nikon E600 microscope that provides a color image in jpg format, with a variable size depending on the desired view field. Although the jpg format is a loss compressed format, but the morphology is preserved and what is of interest to us is the intensity of the dye which has not been significantly changed.

### Methods

Image segmentation based on discontinuity tends to partition an image by detecting isolated points based on an abrupt change in gray levels [9,12]. In the framework of our application, we are interested in segmenting the color images represented by several space colors. In fact, the problem is to detect the actual region boundaries with closed curves.

The output of most existing edge detectors such as canny operator [8,9], can only provide candidates for the regions boundaries, because of the obtained color edges normally discontinuous or over-detected. In this context, color edge detection using statistical features and the threshold technique appears to be an interesting method.

The segmentation method, proposed in this paper, is conceptually different and explores a new strategy. In fact, instead of considering an existing segmentation procedure, the proposed technique rather explores the benefit of combining several approaches.

This method is a hybrid image segmentation technique which integrates both the results of the statistical method and the automatic threshold technique, in which the statistical features are used as the initial seed for the threshold procedures.

In this paper, instead of using the simple edge detectors, a statistical feature extracted from the co-occurrence matrix is used. The co-occurrence matrix is computed starting from a sliding window centered on the pixels of the original image. Note that the concept of co-occurrence matrix [13] was defined to express the image properties related to the second order statistics.

Meaningfully, the proposed method is divided into two stages. At the first stage, the statistical features are extracted from the co-occurrence matrix to construct a new image called the attribute image [13], and then, in the second stage, the threshold technique is then used to obtain the final segmentation results.

**Figure 1:**
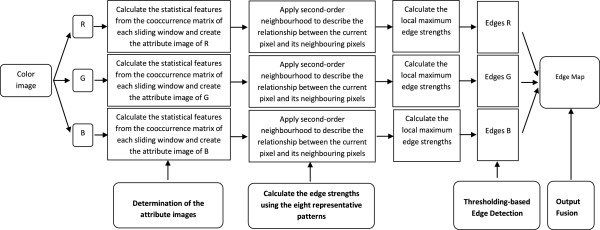
Flowchart of the proposed method.

This technique leads the user to find an optimal segmented image with closed region boundaries with accurate results much better than using the simple edge detectors [8] or other existing techniques [10,11]. The main steps of the proposed detector are depicted in the flowchart shown in Figure 1.

### Edge detection

In this application, instead of implementing a simple edge detector, the statistical features extracted from the co-occurrence matrix for identifying the relationship between the pixels of an image are used. An improved color-edge detector based on the statistical features and an automatic threshold technique is developed.

The gray-level co-occurrence matrix (GLCM) [13], is largely related to the appearance frequency of couples of pixels from an image. It contains significant information which improves the discrimination classes of an image. Hence, it plays an important role in image segmentation. Here, the co-occurrence matrix is defined as the appearance frequency of pixel couples separated by a distance *d* in a particular direction *θ* and it is also called the spatial information dependence method.

Assume *g*_*xy*_ is the intensity of a pixel *p*_*xy*_ at the location (*x, y*) in an (*M* × *N*) image, *w*_*xy*_ is a size (*t* × *t*) window centred at (*x*, *y*) for the computation of co-occurrence matrix.

The window (*w*_*xy*_) is the local regions where the spatial information dependence method is calculated. Thus, the size of the sliding window affects the computation of the co-occurrence matrix, in other words, affects the computation of the statistical features.

In principle, the window should be big enough to allow sufficient information to compute the statistical features extracted from the co-occurrence matrix. On the other side, a large window is time consuming to be processed. Empirically, a window is chosen for computing the co-occurrence matrix.

The window *w*_*xy*_ is the local region where the spatial information dependence method is calculated. So, the co-occurrence matrix describes the appearance frequency of a pixel couples within a local region. Then, the relation *R* is calculated for each window (*w*_*xy*_) as follows:

(1a) $$\begin{array}{l} \mathrm{Cooc}\left(i,j,R\right)=\text{card}\;\left\{\begin{array}{c} \left(\left(x,y\right),\left(x^{ʹ},y^{ʹ}\right)\right)\in D,\text{checking}\;R\left(d,\theta \right)\\ I\left(x,y\right)=i,I\left(x^{ʹ},y^{ʹ}\right)=j\; \end{array}\right)\\ \;=\sum_{i=1}^{N_{c}}\sum_{i=1}^{N_{c}}\left\{\begin{array}{c} 1\;\text{if}\;I\;\left(x,y\right)=i\;\text{and}\;I\;\left(x^{ʹ}=x+d_{x},y^{ʹ}=y+d_{y}\right)=j\\ 0\;\text{otherwise}. \end{array}\right) \end{array}$$

Each element of *Cooc (i, j, R)* represents the number of pixels couple (*i*, *j*), which denotes how often a pixel with gray-level (gray scale intensity) value *i* occurs horizontally adjacent to a pixel with the value *j* in the image. *R*(*d*, *θ*) is the space relation of the two pixels with *d* the distance between the two pixels and *θ* = {0*°*, 45*°*, 90*°*, 135*°*} the orientation of the two pixels versus the horizontal.

Here, the offset *(d*_*x*_*, d*_*y*_*)*, is specifying the distance between the pixel-of-interest and its neighbour. Note that the offset *(d*_*x*_*, d*_*y*_*)* parameterization makes the co-occurrence matrix sensitive to rotation. Choosing an offset vector, such as the rotation of the image is not equal to *180* degrees, will result in a different co-occurrence matrix for the same (rotated) image.

This can be avoided by forming the co-occurrence matrix using a set of offsets sweeping through 180 degrees at the same distance parameter *d* to achieve a degree of rotational invariance (i.e., [0, *d*] for 0°: Cooc horizontal, [*-d,d*] for 45°: Cooc right diagonal, [*-d,*0] for 90°: Cooc vertical, and [*-d,-d*] for 135°: Cooc left diagonal).

In fact, the features are generated by calculating the features for each one of the co-occurrence matrices obtained by using the directions 0°, 45°, 90°, and 135°, then averaging these four values. The symbol *d*, represents the parameter distance, it can be assigned the value one or higher. In general, *d* value is set to 1 as the parameter distance. Consequently, the co-occurrence matrix allows evaluating locally the region contents of the image, this allows the detection of changes in the local statistics of the image.

Formally, for angles quantized to 45^*°*^ intervals the unnormalized frequencies are defined by:

(1b) $$\begin{array}{l} \mathrm{Cooc}\left(i,j,d,0^{{}^{\circ}}\right)=\text{card}\;\left\{\begin{array}{c} \left(\left(x,y\right),\left(x^{ʹ},y^{ʹ}\right)\right)\in D,\left|\left(x-x^{ʹ}=0,\left|y-y^{ʹ}\right|=d\right)\right),\\ I\left(x,y\right)=i,I\left(x^{ʹ},y^{ʹ}\right)=j\; \end{array}\right)\\ \mathrm{Cooc}\left(i,j,d,45^{{}^{\circ}}\right)=\text{card}\;\left\{\begin{array}{c} \left(\left(x,y\right),\left(x^{ʹ},y^{ʹ}\right)\right)\in D,\left|\left(x-x^{ʹ}=d,y-y^{ʹ}=-d\right)\right),\text{or}\left(x-x^{ʹ}=-d,y-y^{ʹ}=d\right)\;\\ I\left(x,y\right)=i,I\left(x^{ʹ},y^{ʹ}\right)=j\; \end{array}\right)\\ \mathrm{Cooc}\left(i,j,d,90^{{}^{\circ}}\right)=\text{card}\;\left\{\begin{array}{c} \left(\left(x,y\right),\left(x^{ʹ},y^{ʹ}\right)\right)\in D,\left|\left(\;\left|x-x^{ʹ}\right|=d,y-y^{ʹ}=0\right)\right),\\ I\left(x,y\right)=i,I\left(x^{ʹ},y^{ʹ}\right)=j\; \end{array}\right)\\ \mathrm{Cooc}\left(i,j,d,135^{{}^{\circ}}\right)=\text{card}\;\left\{\begin{array}{c} \left(\left(x,y\right),\left(x^{ʹ},y^{ʹ}\right)\right)\in D,\left|\left(x-x^{ʹ}=d,y-y^{ʹ}=d\right)\right),\text{or}\left(x-x^{ʹ}=-d,y-y^{ʹ}=-d\right),\\ I\left(x,y\right)=i,I\left(x^{ʹ},y^{ʹ}\right)=j\; \end{array}\right) \end{array}$$

where *card* denotes the number of elements in the set.

**Figure 2:**
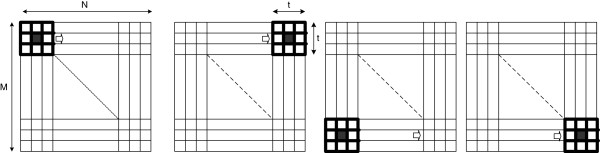
The adaptive sliding window from left to right and top to bottom on an (M × N) image.

**Figure 3:**
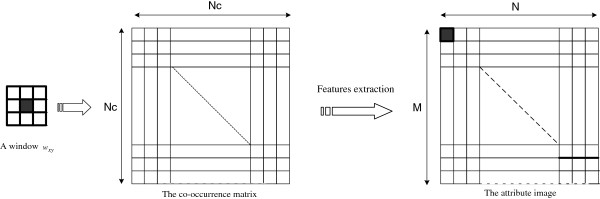
Features extraction from the co-occurrence matrix using a sliding window.

In our application, the statistical features are extracted from the co-occurrence matrix computed from a sliding window (*w*_*xy*_) centred on each pixel *p*_*xy*_ of the original image. The spatial scanning order of an image is performed pixel by pixel from left to right and from top to bottom, (see Figure 2). Hence, this technique is used to calculate the new image called the attribute image. As shown in Figure 3, the value of the attribute image *Ia(x, y)* at the location (*x*, *y*) contains the values of the attribute extracted from the co-occurrence matrix computed from a sliding window (*w*_*xy*_) centred on each pixel *p*_*xy*_ of the original image.

Consequently, several attributes may be extracted from the co-occurrence matrix such as: Mean (*Mean*), diagonal moment *(DM)*, contrast *(Cont)*, energy *(Ener)*, directivity *(Direc)*, entropy *(Entr)*, opposite differential moment *(ODM)*, variance *(Var)*, etc.

Let *Cooc(i, j)* represents the occurring frequency of each pixels couple. It is obtained by calculating how often a pixel with gray-level (gray scale intensity) value *i* occurs horizontally adjacent to a pixel with the value *j* in the *w*_*xy*_ window, *N*_*c*_ is the maximal gray level pixel value of the sliding window *w*_*xy*_.

Among the statistical attributes used in our application, one can define: the diagonal moment (DM), the directivity (Direc), the energy (Ener) and the opposite differential moment (ODM), which are given respectively by the following equations:

(2) $$\mathrm{DM}=\overset{\mathrm{Nc}}{\underset{i=1}{\sum }}\overset{\mathrm{Nc}}{\underset{j=1}{\sum }}{\left(\frac{1}{2}\;\left(\left|i-j\right|\right)\mathrm{Cooc}\left(i,j\right)\right)}^{\frac{1}{2}}$$

(3) $$\mathrm{Direc}=\sum_{i=1}^{\mathrm{Nc}}\sum_{j=1}^{\mathrm{Nc}}\mathrm{Cooc}\left(i,i\right)$$

(4) $$\mathrm{Ener}={\overset{\mathrm{Nc}}{\underset{i=1}{\sum }}\overset{\mathrm{Nc}}{\underset{j=1}{\sum }}\left[\mathrm{Cooc}\left(i,j\right)\right]}^{2}$$

(5) $$\mathrm{ODM}=\sum_{i=1}^{\mathrm{Nc}}\sum_{j=1}^{\mathrm{Nc}}\frac{\mathrm{Cooc}\left(i,j\right)}{1+{\left(i-j\right)}^{2}}$$

where (*N*_*c*_ × *N*_*c*_) is the size of the co-occurrence matrix. The size of the co-occurrence matrix is variable according to the maximum value of the intensity in each window.

Each component of a color image may be taken as a two-dimensional (2*D*) light-intensity function *I*(*x*, *y*), which contains (*M* × *N*) pixels, each with a value of brightness, i.e., grey level, from 0 to Ng. Grey level *0* is the darkest and grey level *Ng* is the brightest. In our study, the task of edge extraction is to classify the pixels into two opposite classes namely edge and non edge classes.

The discontinuity is a measure of abrupt changes in gray levels of pixels *p*_*xy*_, i.e. the discontinuity is described by its edge value, and could be obtained by applying edge detectors to the corresponding region. In the literature, there exist many different edge operators: Sobel, Canny, Derish, Laplace, etc. [8,9], but their functions and performances are not the same. However, most existing edge detectors consider only the horizontal and vertical edge patterns and none of the proposed operators are fully satisfactory in real world use.

**Figure 4:**
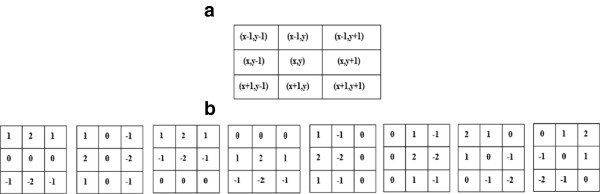
**Spatial relationship between the current pixel (x,y) and its neighbors in the blocs*W***_***xy***_**. (a)** The neighborhood N(x,y) of the current pixel (x,y), **(b)** weight coefficients for calculating the (ES1, ES2, ES3, ES4, ES5, ES6, ES7 and ES8) edge strengths of potential edge patterns.

To overcome this problem, the second-order neighborhood is used to describe the relationship between the current pixel and its neighboring pixels in each attribute image to be combined, as shown in Figure 4(a). An edge may pass through the second neighborhood of a pixel in one of the eight representative patterns shown in Figure 4(b).

These Edge Strengths (ES1, ES2, ES3, ES4, ES5, ES6, ES7 and ES8) are computed as a weighted sum of the pixel values in the neighbourhood *N*(*x*, *y*) of the current pixel (x, y), as shown in Figure 4(a), where the weight coefficients are given in Figure 4(b).

For a given pixel, the edge strengths ES1, ES2, ES3, ES4, ES5, ES6, ES7 and ES8, introduced by the edge patterns shown in Figure 4(b), are computed using the digital convolution of the two-dimensional matrices whose coefficients are the elements of *W*_*xy*_ and each pattern shown in Figure 4(b). These edge strengths are defined respectively as:

(6) $$\begin{array}{l} \mathrm{ES}1\left(x,y\right)=I_{a}\left(x-1,y-1\right)+2I_{a}\left(x-1,y\right)\\ \;+I_{a}\left(x-1,y+1\right)-I_{a}\left(x+1,y-1\right)-2I_{a}\left(x+1,y\right)-I_{a}\left(x+1,y+1\right) \end{array}$$

(7) $$\begin{array}{l} \mathrm{ES}2\left(x,y\right)=I_{a}\left(x-1,y-1\right)-I_{a}\left(x-1,y+1\right)+2I_{a}\left(x,y-1\right)-2I_{a}\left(x,y+1\right)\\ \;+I_{a}\left(x+1,y-1\right)-I_{a}\left(x+1,y+1\right) \end{array}$$

(8) $$\begin{array}{l} \mathrm{ES}3\left(x,y\right)=I_{a}\left(x-1,y-1\right)+2I_{a}\left(x-1,y\right)\\ \;+I_{a}\left(x-1,y+1\right)-I_{a}\left(x,y-1\right)-2I_{a}\left(x,y\right)-I_{a}\left(x,y+1\right) \end{array}$$

(9) $$\begin{array}{l} \mathrm{ES}4\left(x,y\right)=I_{a}\left(x,y-1\right)+2I_{a}\left(x,y\right)\\ \;+I_{a}\left(x,y+1\right)-I_{a}\left(x+1,y-1\right)-2I_{a}\left(x+1,y\right)-I_{a}\left(x+1,y+1\right) \end{array}$$

(10) $$\begin{array}{l} \mathrm{ES}5\left(x,y\right)=I_{a}\left(x-1,y-1\right)-I_{a}\left(x-1,y\right)+2I_{a}\left(x,y-1\right)-2I_{a}\left(x,y\right)\\ \;+I_{a}\left(x+1,y-1\right)-I_{a}\left(x+1,y\right) \end{array}$$

(11) $$\begin{array}{l} \mathrm{ES}6\left(x,y\right)=I_{a}\left(x-1,y\right)-I_{a}\left(x-1,y+1\right)+2I_{a}\left(x,y\right)-2I_{a}\left(x,y+1\right)\\ \;+I_{a}\left(x+1,y\right)-I_{a}\left(x+1,y+1\right) \end{array}$$

(12) $$\begin{array}{l} \mathrm{ES}7\left(x,y\right)=2I_{a}\left(x-1,y-1\right)+I_{a}\left(x-1,y\right)\\ \;+I_{a}\left(x,y-1\right)-I_{a}\left(x,y+1\right)-I_{a}\left(x+1,y\right)-2I_{a}\left(x+1,y+1\right) \end{array}$$

(13) $$\begin{array}{l} \mathrm{ES}8\left(x,y\right)=I_{a}\left(x-1,y\right)+2I_{a}\left(x-1,y+1\right)-I_{a}\left(x,y-1\right)\\ \;+I_{a}\left(x,y+1\right)-2I_{a}\left(x+1,y-1\right)-I_{a}\left(x+1,y\right) \end{array}$$

where *I*_*a*_(*x*-1,*y*-1) indicates the value of the pixel at (*x*-1,*y*-1) in the attribute image *I*_*a*_. The local maximum edge strength of pixel (*x, y*), *LMES(x,y)* is defined as the maximum of the eight edge strengths:

(14) $$\mathrm{LMES}\left(x,y\right)=\max\left\{\mathrm{ES}1,\mathrm{ES}2,\mathrm{ES}3,\mathrm{ES}4,\mathrm{ES}5,\mathrm{ES}6,\mathrm{ES}7,\mathrm{ES}8\right\}$$

Given the optimal thresholds: *(T*_*R*_*, T*_*G*_*and T*_*B*_*)* respectively for attribute images of the three primitive colors Red, Green and Blue, the (E_R_, E_G_ and E_B_) functions classify the pixel on the Red, Green and Blue components, into two opposite classes: edge pixels versus non edge pixels, as:

(15) $$E_{R}\left(x,y\right)=\left\{\begin{array}{c} 1,\mathrm{edge}\;\mathrm{pixel}\;\mathrm{if}\;{\mathrm{LMES}}_{R}\left(x,y\right)\geq T_{R}\\ 0,\mathrm{non}-\mathrm{edge}\;\mathrm{pixel}\;\mathrm{if}\;{\mathrm{LMES}}_{R}\left(x,y\right)<T_{R} \end{array}\;\right\}$$

(16) $$E_{G}\left(x,y\right)=\left\{\begin{array}{c} 1,\mathrm{edge}\;\mathrm{pixel}\;\mathrm{if}\;{\mathrm{LMES}}_{G}\left(x,y\right)\geq T_{G}\\ 0,\mathrm{non}-\mathrm{edge}\;\mathrm{pixel}\;\mathrm{if}\;{\mathrm{LMES}}_{G}\left(x,y\right)<T_{G} \end{array}\;\right\}$$

(17) $$E_{B}\left(x,y\right)=\left\{\begin{array}{c} 1,\mathrm{edge}\;\mathrm{pixel}\;\mathrm{if}\;{\mathrm{LMES}}_{B}\left(x,y\right)\geq T_{B}\\ 0,\mathrm{non}-\mathrm{edge}\;\mathrm{pixel}\;\mathrm{if}\;{\mathrm{LMES}}_{B}\left(x,y\right)<T_{B} \end{array}\;\right\}$$

The optimal thresholds *T*_*R*_*, T*_*G*_*and T*_*B*_, are automatically determined by the Otsu’s threshold technique, as described in Section 2.2. The pixel *(x,y)* of each primitive color is classified as an edge pixel if its local maximum edge strength of the attribute image *(LMES)* is higher than the optimal threshold determined automatically by the Otsu’s threshold technique, in which case is set to 1. Otherwise, it is classified as a non-edge pixel and is set to 0.

Edge results for the three color components are then integrated through the fusion rule, shown in Eq. 18. Pixel (x,y) is classified as an edge pixel if it is so classified by at least one of its three color components, in which case E(x, y) is set to 1. Otherwise, it is classified as a non-edge pixel and *E(x, y)* is set to 0. The joint edge is calculated according to the following formula:

(18) $$E\left(x,y\right)=\left\{\begin{array}{c} 1,\text{edge}\;\text{pixel},\mathrm{if}\;E_{R}\left(x,y\right)=1\;\cup \;E_{G}\left(x,y\right)=1\;\cup \;E_{B}\left(x,y\right)=1\;\\ 0,\text{nonedge}\;\text{pixel},\text{otherwise} \end{array}\right)$$

### Otsu’s Threshold method

Generally, it is quite important to effectively determine a threshold value for a gray-level image when extracting the edges, i.e., to extract objects of interest from their background.

In this study, the optimal threshold can be readily selected.

by the Otsu threshold technique [14,15]. The technique is shown to be highly efficient for the two-class data classification problem.

To illustrate this, let the local maximum edge strength of pixels have range [0, *L*]. In an input image, let’s assume that there are f_i_ pixels whose local maximum edge strength has the value *i*, with *i* ∈ [0, *L*].

If an image can be divided into two classes, edge and non-edge classes, by a threshold at level t, where edge class consists of the local maximum edge strength features from 0 to t, and non-edge contains the other local maximum edge strength features with *t* + 1 to *L*, then the probability distributions for the edge and non-edge pixel classes can be defined, respectively.

The probability for the pixels having the local maximum edge strength features in range 0 ≤ i ≤ L, is defined by:

(19) $$p_{i}=\frac{f_{i}}{N}$$

Where $N=\sum_{k=0}^{L}f_{k}$ indicates the total number of pixels.

The cumulative probability (w_1_ and w_2_) for edge and non- edges classes respectively, are given by:

(20) $$w_{1}=\sum_{i=0}^{t}p_{i}$$

(21) $$w_{2}=\sum_{i=t+1}^{L}p_{i}$$

The means of local maximum edge strength features (μ_1_, μ_2_) for edge and non-edges classes, respectively, are defined by:

(22) $$\mu_{1}=\sum_{i=0}^{t}\frac{{\mathrm{ip}}_{i}}{w_{1}}$$

(23) $$\mu_{2}=\sum_{i=t+1}^{L}\frac{{\mathrm{ip}}_{i}}{w_{2}}$$

The optimal threshold T is selected for performing the non-edge and pixel classification satisfying the following criterion function:

(24) $$T=\arg_{0\leq t\leq L}\max\{\sigma_{B}^{2}\left(t\right)\sigma_{B}^{2}=w_{1}{\left(\mu_{1-}\mu_{T}\right)}^{2}+w_{2}{\left(\mu_{2-}\mu_{T}\right)}^{2}$$

where $\mu_{T}=\sum_{i=0}^{L}{\mathrm{ip}}_{i}$ is the total mean of the local maximum edge strength features.

As a result, an optimal bi-level threshold can be readily selected by the Otsu’s threshold method by maximizing the between-class variance of the two classes. The major steps of the proposed detector are shown in Figure 1.

## Results and discussion

### Dataset

**Figure 5:**
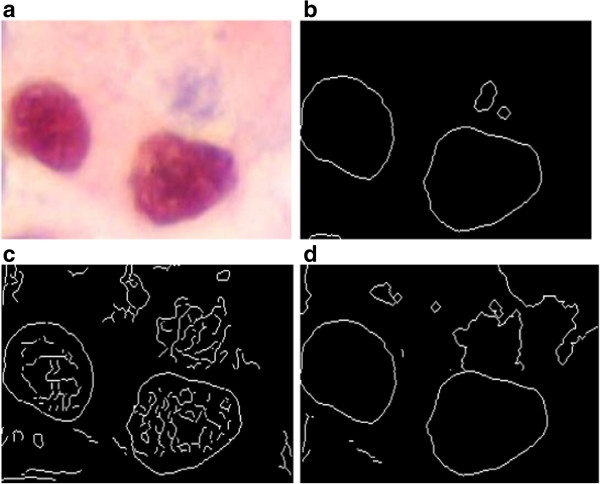
**Edge detection results on a color image. (a)** Original image (256 × 256 × 3) with gray level spread on the range [0, 255], **(b)** Red resulting image by our technique, **(c)** Green resulting image by our technique, **(d)** Blue resulting image by our technique.

**Figure 6:**
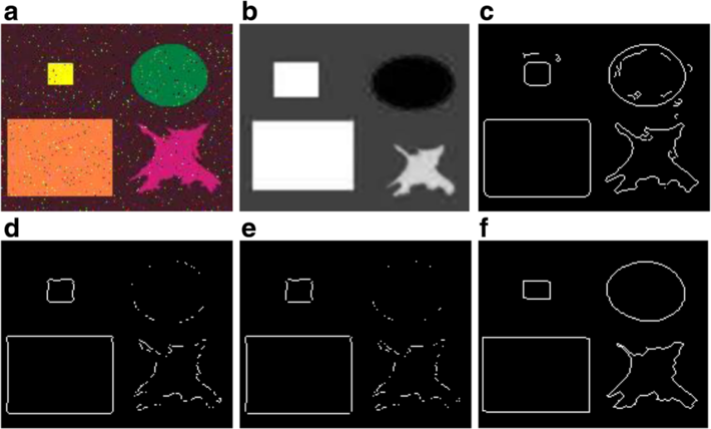
**Comparison of the proposed segmentation method with other existing methods on a synthetic image. (a)** Original image with RGB representation (256 × 256 × 3), disturbed with a “salt and pepper” noise, **(b)** variance image, **(c)** edges obtained by the Prewitt operator, **(d)** Edges obtained by the Canny operator, **(e)** Edges obtained by the Sobel operator, **(f)** Edges obtained by our technique.

**Figure 7:**
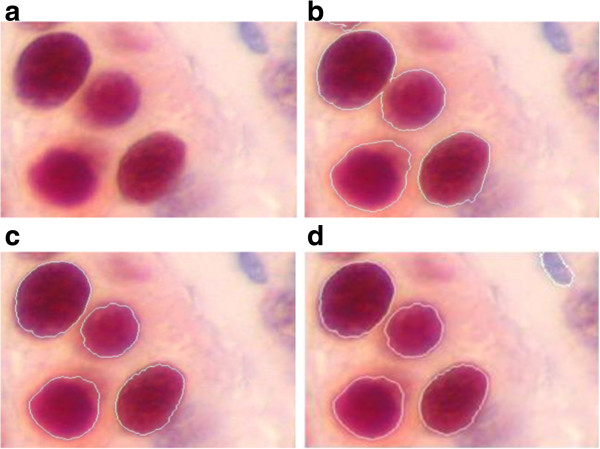
**Edge detection performance comparisons for cells image. (a)** original image, **(b)** edges obtained by the proposed technique, using the first order statistical features **(c)** edges obtained by the proposed technique, using the second order statistical features, **(d)** the reference edge pixels. *(The various medical images used in this paper are provided with permission from Cancer Service, Salah Azaiez Hospital, Bab Saadoun, Tunis, Tunisia).*

**Figure 8:**
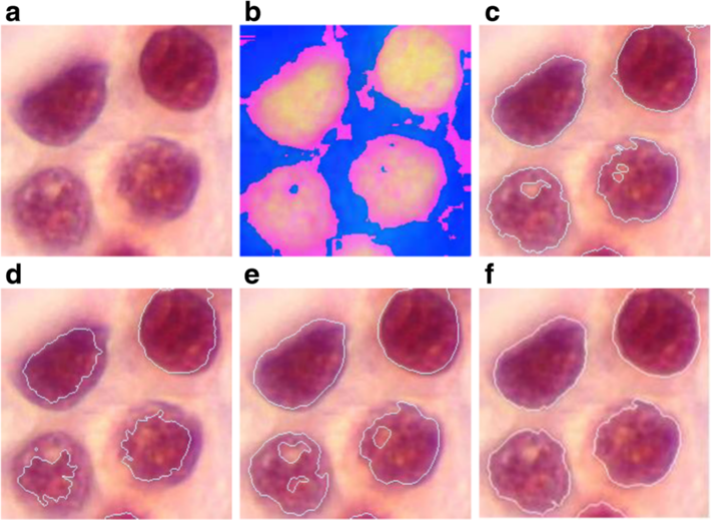
**Comparison of the proposed segmentation method with other existing methods on a medical image (2 classes, several cells). (a)** original image with RGB representation (256 × 256 × 3), **(b)** Original image with HSI representation, **(c)** segmentation based on CED_VS algorithm, **(d)** segmentation based on CED_HSI algorithm **(e)** segmentation based on VS_CED algorithm, **(f)** segmentation based on the proposed method.

In this section, a large variety of color images is employed in our experiments. Some experimental results are shown in Figures 5, 6, 7 and 8.

The images originally are stored in RGB format. Each of the primitive color (red, green and blue) takes 8 bits and has the intensity range from 0 to 255. The labeling of the original image is generated by the user based on the image used for segmentation.

**Figure 9:**
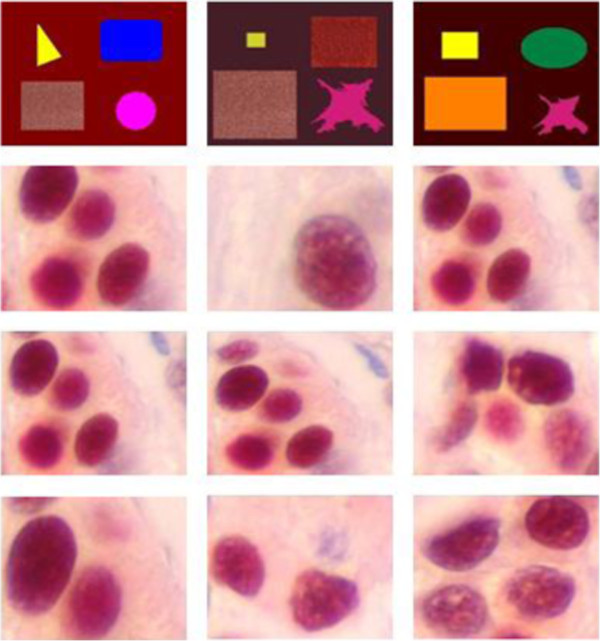
**Data set used in the experiment. Twelve were selected for a comparison study.** The patterns are numbered from 1 through 12, starting at the upper left-hand corner.

In order to illustrate the method presented in the previous section, several segmentation results of medical and synthetic color images are given. The used images data base is shown in Figure 9.

### Comparisons

In fact, to evaluate the efficiency and accuracy of the proposed edge extraction method, the results are compared versus existing methods, as described earlier. The efficiency evaluations for these different methods are carried out on the Matlab software 7.1.

First the segmentation results in RGB color space by applying the proposed method to red, green, and blue color features, respectively, are presented. Figure 5(a) gives the original image. The results of R, G and B components by the proposed method are shown in Figure 5(b),(c) and (d) respectively. In this case, some obvious edges in the color image are missed if the edge detector is performed on each component image (R, G and B) independently.

This shows the lack of information when using only one information source and may be explained by the high degree of correlation among of the three components of the RGB color space. Hence, it demonstrates the necessity of the merging process.

Also, to evaluate the performance of the proposed color-edge detector, we tested many color synthetic images.

Figure 6(a) gives the (N × M) synthetic image where a “salt and pepper” noise of D density was added. This affects approximately (D × (N × M)) pixels. Figure 6(b) shows the variance image.

The results obtained by the Prewitt, Canny, Sobel and Laplacian edge detectors are given in Figure 6(c),(d),(e), respectively. Figure 6(f) shows the extracted edges by performing our edge detector on the variance image.

In fact, some obvious edges in the image are missed and some incorrectly classified pixels are presented if only the simple edges detectors are performed on the noisy image.

Note that our edge detector can provide more potential edges as compared with the other detectors under the same conditions.

Accordingly, the experimental results indicate that the proposed edge detector can provide more accurate results, see Figure 6(f).

To evaluate the performance of the proposed method, its accuracy was recorded. Regarding the accuracy, Table 1 lists the segmentation sensitivity of the different methods for the dataset used in the experiment.

**Table 1:** **Segmentation sensitivity from Prewitt, Canny, Sobel operator and the proposed method for the data set shown in Figure **9

	P ***rewitt operator***	***Canny operator***	***Sobel operator***	***Proposed technique***
	***Sensitivity segmentation (%)***			
***Image 1***	*85.32*	*87.44*	*86.15*	*92.37*
***Image 2***	*64.52*	*88.96*	*85.54*	*96.46*
***Image 3***	*74.98*	*87.27*	*86.37*	*95.76*
***Image 4***	*88.53*	*95.60*	*94.31*	*95.45*
***Image 5***	*76.79*	*91.17*	*77.28*	*97.94*
***Image 6***	*74.28*	*87.72*	*93.46*	*96.48*
***Image 7***	*76.46*	*96.46*	*85.47*	*97.24*
***Image 8***	*64.18*	*88.25*	*91.45*	*94.35*
***Image 9***	*74.73*	*87.44*	*93.73*	*96.43*
***Image 10***	*66.17*	*79.76*	*92.01*	*95.76*
***Image 11***	*47.46*	*69.28*	*77.19*	*88.25*
***Image 12***	*56.49*	*67.16*	*81.72*	*92.43*

The segmentation sensitivity [16,17], is determined as follows:

(25) $$\mathrm{Sen}\left(\%\right)=\frac{N_{\mathrm{pcc}}}{N\times M}\times 100$$

with: *Sen*(*%*), *N*_*pcc*_, *N* × *M* denote respectively the segmentation sensitivity (%), the number of correctly classified pixels and the dimension of the image.

Referring to the segmentation sensitivity given in Table 1, one can observe that 25.02, 12.73, 13.63 and 04.24 of pixels were missegmented in Figure 6(c),(d),(e) and (f), respectively. Comparing Figure 6(c),(d) and (e) with (f), it is clear that the resulting image by the proposed edge detector is much better than the one by the Prewitt, Canny and Sobel operators. In fact, the different regions are correctly classified in Figure 6(f) which is not the case in Figure 6(c), (d) and (e).

Another evaluation criterion is used to measure quantitatively the detection quality of the signal corresponding to the edge of the objects. This criterion is based on the determination of the probability of correct detection and false detection. In this case, reference edge pixels are necessary (see Figure 7(d)). This criterion resides in the comparison between labels of the obtained edge pixels and the reference edge pixels.

In the case where two objects are presented in the reference image R, R = R_1_ ∪ R_2_, the segmentation of two objects can be regarded as a classification problem. Consequently, the result can be measured as the probability of correct classification (P_C_) and false classification (P_f_), which are defined respectively by:

(26) $$P_{c}=\frac{N_{1c}}{N_{1r}}$$

(27) $$P_{f}=\frac{N_{1f}}{N_{2r}}\;$$

where N_1r_ and the N_1r_ is the number of pixel in the reference R_1_ and R_2_, respectively. N_1C_ is the number of correctly classified pixels relative with R_1_. N_1f_ is the number of pixels which belong to R_2_ and incorrectly classified in R_1_. These criteria were employed by Dou et al. [18], to evaluate the effectiveness of the segmentation method in the case of abnormal tissue. In our application, these criteria are used to evaluate the proposed segmentation method in the case of color images of breast cancer cells.

Suppose R_1_ the cells pixels and R_2_ the background, thus (P_f_) represents the probability that a background pixel is marked as a cell pixel. (P_C_) represents the probability that a cell pixel is actually marked as a cell pixel.

The process for obtaining the correct classified pixels is not a manual process; thus a software based reference image is run. It consists in a small program which compares the labels of the obtained pixels and the reference pixels as shown in Figure 7(d). The correctly classified pixel denotes a pixel with a label equal to its corresponding pixel in the reference image.

Since the proposed method is based on the second order statistical feature and the threshold technique, we perform a comparison with several other well-known first order statistical features combined with threshold technique (FSFT) [5].

Figure 7 shows a comparison of the results obtained by the proposed method using the first and the second order statistical features, applied to color cell images (which is a challenging problem in this field).

They correspond, respectively, to Figure 7(b) and (c). In fact, the cells are correctly localized in Figure 7(c) which is not the case in Figure 7(b).

This demonstrates the superiority of combining the second order statistical features and the threshold technique for edges detection.

The performance of the proposed method is quite acceptable. Referring to probability of correct classification (P_C_) and false classification (P_f_) given in Table 2, we can observe in Figure 7 that the probability of correct classification obtained by the proposed method (81.41%) is higher than that obtained by FSFT method (65.95%).

**Table 2:** **The probability of correct classification and false classification from the FSFT method and the proposed method for the data set shown in Figure**9

	***Probability of correct classification*** (P_C_) ***and false classification*** (P_***f***_)			
	***FSFT method***		***Proposed technique***	
	(P_C_ ***)***	(P_***f***_)	(P_C_)	(P_***f***_)
*Image 1*	*0.5656*	*0.27*	*0.6196*	*0.13*
*Image 2*	*0.6187*	*0.39*	*0.6778*	*0.21*
*Image 3*	*0.6434*	*0.19*	*0.7081*	*0.15*
*Image 4*	*0.6074*	*0.35*	*0.7117*	*0.11*
*Image 5*	*0.6197*	*0.33*	*0.7786*	*0.13*
*Image 6*	*0.6595*	*0.29*	*0.8141*	*0.17*
*Image 7*	*0.7735*	*0.17*	*0.8973*	*0.14*
*Image 8*	*0.6855*	*0.26*	*0.8464*	*0.21*
*Image 9*	*0.5790*	*0.38*	*0.7081*	*0.17*
*Image 10*	*0.7592*	*0.18*	*0.8979*	*0.15*
*Image 11*	*0.6009*	*0.35*	*0.7441*	*0.19*
*Image 12*	*0.6892*	*0.26*	*0.8027*	*0.21*

Its indicator corresponding to the probability of false classification (17%) is also lower than the FSFT method (29%).

Moreover, this demonstrates that the simple edge detectors are instable in noisy environments and provides edges normally discontinuous or badly detected.

In fact, errors were largely reduced when exploiting simultaneously the second order statistical features and threshold techniques for color edges extraction.

Consequently, the proposed method allows to obtain a binary mask (see Figure 7(c)), which can be used to locate the edge of the cells.

Also let us compare the performance of the proposed algorithm to those in other published reports that have been applied to color images. These include Trahanias et al. [19,20] and Carron et al. [21]. The segmentation results are shown in Figure 8.

Figures 8(c), (d), (e) and (f) show the final segmentation results obtained from the CED_VS algorithm [19], the CED_HSI algorithm [21], the VS_CED method [20] and the proposed algorithm, respectively. Comparing Figure 8(c), (d), (e) and (f), one can observe that the two regions are correctly segmented in Figure 8(f), e.g. the cell is much better segmented in (f) than those in (c), (d) and (e), also the first, the second and the third images contain some holes in the cell and some pixels were incorrectly segmented. These do not exist in the correctly segmented image, but using the proposed algorithms, only a few singularity points are left in the final image as shown in Figure 8(f).

**Figure 10:**
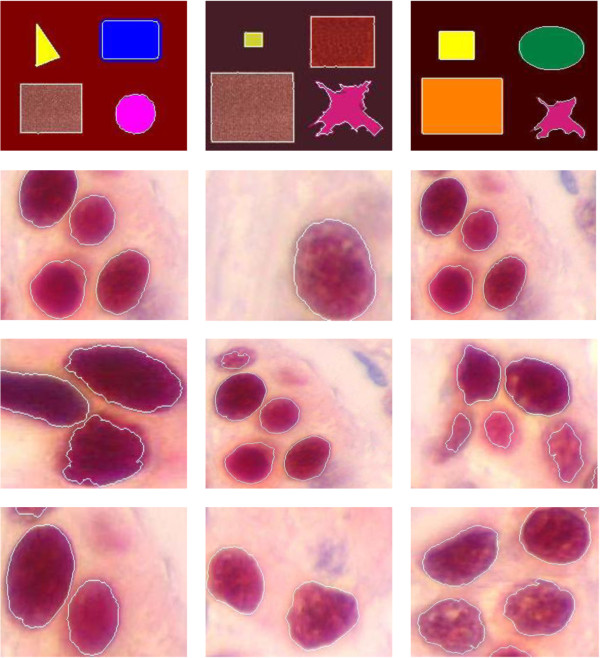
Color edges detection.

Figure 10 illustrates the final results for the used images data base (Figure 9), where the edges in white color are superimposed on the original images.

## Conclusion

In this paper, we have proposed a new method for color edges detection based on statistical features and threshold technique. In the first phase, the edges are identified in each attribute image of the three primitive colors via a threshold operation. Then, edge results for the three color components are integrated through the fusion rule.

Instead of considering and elaborating and better designed segmentation model of natural and textured images, the proposed technique rather explores the possible alternative of combining two segmentation techniques in order to get a good consistency edges.

The results obtained show the generic and robust character of the method in the sense that the spatial neighborhood information is introduced to take into account the spatial correlation between a pixel couples from an image that exist in any physical images. Also, this technique can accurately detect the edges and suppress the impact of the noise on the results, while the edge has a good consistency. Extensive testing results have shown great potential on the proposed method. It can be useful for color edges detection.

## Competing interests

The authors declare that they have no competing interests.

## Authors’ contributions

SBC and FF performed the scientific computation and experiments. Both authors read and approved the final manuscript.
